# Episode-of-Care Costs for Revision Total Joint Arthroplasties by Decadal Age Groups

**DOI:** 10.3390/geriatrics6020049

**Published:** 2021-05-11

**Authors:** Christopher Fang, Nicholas Pagani, Matthew Gordon, Carl T. Talmo, David A. Mattingly, Eric L. Smith

**Affiliations:** 1 New England Baptist Hospital, 125 Parker Hill Ave, Boston, MA 02120, USA; cfang@nebh.org (C.F.); ctalmo@nebh.org (C.T.T.); dmatting@nebh.org (D.A.M.); 2 Department of Orthopaedic Surgery, Tufts Medical Center, 800 Washington St, Boston, MA 02111, USA; nicholas.pagani@gmail.com (N.P.); mgordon1@tuftsmedicalcenter.org (M.G.)

**Keywords:** older patients, revision total hip arthroplasty, revision total knee arthroplasty, octogenarians, nonagenarians, TDABC, costs

## Abstract

The demand for revision total joint arthroplasties (rTJAs) is expected to increase as the age of the population continues to rise. Accurate cost data regarding hospital expenses for differing age groups are needed to deliver optimal care within value-based healthcare (VBHC) models. The aim of this study was to compare the total in-hospital costs by decadal groups following rTJA and to determine the primary drivers of the costs for these procedures. Time-driven activity-based costing (TDABC) was used to capture granular hospital costs. A total of 551 rTJAs were included in the study, with 294 sexagenarians, 198 septuagenarians, and 59 octogenarians and older. Sexagenarians had a lower ASA classification (2.3 vs. 2.4 and 2.7; *p* < 0.0001) and were more often privately insured (66.7% vs. 24.2% and 33.9%; *p* < 0.0001) as compared to septuagenarians and octogenarians and older, respectively. Sexagenarians were discharged to home at a higher rate (85.3% vs. 68.3% and 34.3%; *p* < 0.0001), experienced a longer operating room (OR) time (199.8 min vs. 189.7 min and 172.3 min; *p* = 0.0195), and had a differing overall hospital length of stay (2.8 days vs. 2.7 days and 3.6 days; *p* = 0.0086) compared to septuagenarians and octogenarians and older, respectively. Sexagenarians had 7% and 23% less expensive personnel costs from post-anesthesia care unit (PACU) to discharge (*p* < 0.0001), and 1% and 24% more expensive implant costs (*p* = 0.077) compared to septuagenarians and octogenarians and older, respectively. Sexagenarians had a lower total in-hospital cost for rTJAs by 0.9% compared to septuagenarians but 12% more expensive total in-hospital costs compared to octogenarians and older (*p* = 0.185). Multivariate linear regression showed that the implant cost (0.88389; *p* < 0.0001), OR time (0.12140; *p* < 0.0001), personnel cost from PACU through to discharge (0.11472; *p* = 0.0007), and rTHAs (−0.03058; *p* < 0.0001) to be the strongest associations with overall costs. Focusing on the implant costs and OR times to reduce costs for all age groups for rTJAs is important to provide cost-effective VBHC.

## 1. Introduction

Approximately 7 million Americans are living with a total hip or knee replacement [1]. As patients live longer and the average age of the U.S. population increases, concomitant growths in the numbers of revision total hip arthroplasties (rTHAs) and total knee arthroplasties (rTKAs) are anticipated. By 2030, over 20% of the U.S. population will be over the age of 65 and a 137% increase in the number of rTHAs and a 601% increase in the number of rTKAs is expected [1,2,3,4]. Between 2010 and 2050, the population of nonagenarians is projected to quadruple [5]. Given these demographic trends, the burden of revision total joint arthroplasties (rTJAs) in these geriatric patients will continue to grow dramatically [6].

rTHAs and rTKAs are more costly and more demanding of provider and institutional resources than primary arthroplasty procedures [7,8,9]. Revision arthroplasty procedures have prolonged operative times, increased blood loss, and higher rates of adverse events in contrast to primary THA/TKA procedures. Age and medical comorbidities have been shown to amplify the risk of complications following revision arthroplasty. Within a national system emphasizing value-based healthcare (VBHC), geriatric patients may be viewed as carrying unacceptably high medical and financial risk for revision surgery in comparison to younger patients, and therefore have limited access to these procedures. As both surgeons and healthcare systems are incentivized to improve outcomes while reducing costs, an improved understanding of how patient age and associated comorbidities impact hospitalization costs and expenditures related to revision arthroplasty is needed.

The primary aim of our study was to compare the cost differences for an rTJA episode of care among age groups by decades. We also sought to determine which factors contribute to hospital costs among these groups. We hypothesized that hospital cost differences would exist between decadal age groups for rTJAs.

## 2. Materials and Methods

### 2.1. Study Design

After internal review board (IRB) approval was obtained (#1629639-1), financial data were retrospectively collected between January 2018 through to May 2020 at our single-specialty orthopedic hospital for patients aged 60–69 years (sexagenarians), 70–79 years (septuagenarians), and 80 years and older (octogenarians and nonagenarians) who underwent either an rTHA or an rTKA. We collected data on patient demographics, including age, American Society of Anesthesiologists (ASA) classification, procedure type, and insurance, as well as discharge disposition, operating room (OR) time, length of stay (LOS), personnel cost of the post-anesthesia care unit (PACU) through to discharge, and implant and total in-hospital costs to compare the differences between decadal age groups. Financial data are presented as indexed values to protect institutional proprietary information.

### 2.2. Time-Driven Activity-Based Costing

Time-driven activity-based costing (TDABC) was used with the help of a third-party, commercial cost-analysis database, Avant-garde Health (Boston, MA, USA), to determine the episode-of-care costs. TDABC is a modern cost-accounting methodology that is designed to improve patient value [10] and allows for analyzing patient-level, granular cost data based on the time spent on specific activities. Total in-hospital costs are the summation of total personnel and supply costs that were directly used in the care of the patient. Supply costs were determined by the actual purchase price. Indirect costs not specific to the patient (e.g., utilities, unused space) were not included in this study.

### 2.3. Statistical Analysis

To compare the characteristics and outcomes between sexagenarians, septuagenarians, and octogenarians and older undergoing rTHAs and rTKAs, chi-square and analysis of variance (ANOVA) tests were used where appropriate for categorical and continuous data, respectively. Continuous quantitative variables are expressed as an average with a standard deviation after a normal distribution was confirmed via ANOVA residual plot analysis. Multivariate linear regression was used to estimate the relationship between the total in-hospital cost and other significant independent variables, such as OR time, implant cost, and length of stay. To protect confidential internal data, standardized beta coefficients and 95% confidence intervals were used to compare the strength of the effect of each individual independent variable to total in-hospital costs. A standardized regression coefficient is calculated by dividing a parameter estimate by the ratio of the sample standard deviation of the dependent variable to the sample standard deviation of the regressor. All statistical analyses were performed using SAS v9.4 (SAS Institute, Cary, NC, USA). Significance was set at 0.05. A post-hoc Bonferroni *p*-value correction was applied and the significance threshold was set to *p* = 0.003 for our multiple procedure group comparisons.

## 3. Results

A total of 551 rTJAs were included in the study (Table 1). Of these procedures, 294 were performed on sexagenarian patients, 198 on septuagenarians, and 59 on octogenarian (*n* = 55) and nonagenarian (*n* = 4) patients (Table 1). On average, sexagenarians had a lower ASA classification (2.3 vs. 2.4 and 2.7; *p* = 0.0014) and were more often privately insured (66.7% vs. 24.2% and 33.9%; *p* < 0.0001) compared to septuagenarians and octogenarians and older, respectively. Sexagenarians underwent a higher percentage of rTKA procedures, but this difference was not significant (*p* = 0.226).

### 3.1. rTHA

A total of 275 (49.9%) of the procedures were rTHAs (Table 2). Among the rTHAs, sexagenarians were more often discharged to home (85.3% vs. 68.3% and 34.3%; *p* < 0.0001) and experienced a longer OR time (199.8 min vs. 189.7 min and 172.3 min; *p* = 0.0195) compared to septuagenarians and octogenarians and older, respectively. Sexagenarians had a longer LOS compared to septuagenarians (2.8 days vs. 2.7 days) but a shorter LOS compared to octogenarians and older (2.8 days vs. 3.6 days; *p* = 0.0086). Sexagenarians also had higher personnel costs from the PACU through to discharge by 1% compared to septuagenarians, but personnel costs were 22% less expensive than octogenarians and older (*p* = 0.0064). The decadal age groups did not differ in implant cost or total in-hospital costs for rTHAs (*p* = 0.845 and 0.618, respectively).

### 3.2. rTKA

A total of 276 (50.1%) of the procedures were rTKAs (Table 3). Among the rTKAs, sexagenarians were more often discharged to home (85.5% vs. 63.8% and 16.7%; *p* < 0.0001) and experienced longer LOS (2.8 vs. 3.4 and 3.8; *p* < 0.0001) compared to septuagenarians and octogenarians and older, respectively. The OR times between the decadal age groups were similar (*p* = 0.23).

Sexagenarians had lower personnel costs from the PACU through to discharge by 16% compared to septuagenarians and 28% compared to octogenarians and older (*p* = 0.0001). The decadal age groups did not differ in implant cost or total in-hospital costs for rTKAs (*p* = 0.214 and 0.24, respectively); however, the octogenarian and older age group had 26% lower implant costs than sexagenarians, which was likely due to the eight lower-cost liner exchange procedures in this group.

### 3.3. rTHA and rTKA

Overall, sexagenarians were more often discharged to home (85.4% vs. 66.2% and 27.1%; *p* < 0.0001), experienced a longer OR time (197.3 min vs. 191.1 min and 173.4 min; *p* = 0.0021), shorter length of stay (2.8 vs. 3.1 and 3.7; *p* < 0.0001), lower personnel costs from the PACU through to discharge (*p* < 0.0001), and had 1% and 24% lower implant costs (*p* = 0.077) compared to septuagenarians and octogenarians and older, respectively (Table 4). rTJAs for sexagenarians were 0.9% less expensive than septuagenarians but 12% more expensive than octogenarians and older, but this was not statistically significant (*p* = 0.185).

The results of the multivariate linear regression analysis of the total in-hospital costs are reported in Table 5. The independent variables explained 99% of the variation in the revision hip and knee TJA in-hospital costs (Table 5). The implant cost was positively associated with the strongest effect (0.88389; *p* < 0.0001) on the total in-hospital cost. The OR time was positively associated with the second-strongest effect (0.12140; *p* < 0.001) and the personnel cost PACU through to discharge was also positively associated with the third-strongest effect (0.11472; *p* = 0.0007) on the total in-hospital cost. The rTHA procedure type was negatively associated with the total in-hospital cost (−0.03058; *p* < 0.0001). Decadal age group was not a significant factor after holding all other variables constant (*p* = 0.19 for septuagenarians and *p* = 0.45 for octogenarians and older).

## 4. Discussion

By 2050, 10% of the United States’ elderly population 65 years and older will be persons 90 years or older [5]. Further, 80.8% of nonagenarians have a disability, which is most commonly related to mobility activities, such as walking. As the population with osteoarthritis treated with TJA continues to rise, an increase in revision arthroplasty procedures in older populations is anticipated. Previously, our institution used a similar methodology to analyze cost differences of primary TJA procedures among octogenarians and nonagenarians [11]. Revision surgeries are more expensive than primary procedures, and as the country moves to value-based payment models, it is important for institutions to understand the costs of revision surgery and which factors increase overall costs. Older patients, such as octogenarians and nonagenarians with increased comorbidity burdens, may be seen as a patient population carrying a high financial risk. Thus, access to indicated revision procedures may be limited for these patients. However, our study showed that total in-hospital costs for octogenarians and nonagenarians undergoing rTJAs were not higher than the costs associated with revision procedures for sexagenarians or septuagenarians. Porter describes value in medicine as the health outcomes achieved per dollar spent [8]. Our findings show that value can still be attained with revision procedures in octogenarians and nonagenarians with comparable amounts of money spent. In order to provide VBHC, a focus on improving outcomes and limiting complications in these older populations is paramount.

A predominant concern when considering revision arthroplasty in elderly patients is patient safety and the risk of perioperative complications based on medical comorbidities. On average, octogenarians and nonagenarians had a higher ASA classification than sexagenarians and septuagenarians. ASA ratings correlate with patient medical comorbidities and can be helpful in predicting perioperative risks and outcomes. Previous studies have looked at outcomes for nonagenarians and octogenarians in revision procedures [12,13,14,15,16,17]. Bovonratwet et al. found revision TKA to be safe in patients aged over 80 years and concluded that octogenarian patients need not be discouraged from revision TKA solely based on their advanced age [14]. Song et al. also demonstrated an rTKA to be a viable option for octogenarians due to satisfactory clinical outcomes, complication rates, and mid-term lifetime implant survival [15]. Parvizi et al. showed that an rTHA can provide substantial clinical benefit to patients over eighty years of age [16]. However, there is additional evidence that an aseptic rTHA may be associated with greater risks in patients 80 years or older compared to younger patients [17].

For rTHAs, nonagenarians and octogenarians require transfusions 33.13% and 24% of the time, respectively [13]. Patients over 80 years of age have been shown to have a higher risk of perioperative mortality, pneumonia, urinary tract infection, and extended length of stay compared to patients under 80 years of age following an rTHA [17]. However, a separate study found medical complication rates for octogenarians following an rTHA to be comparable to younger patients [16]. The authors of the second study also showed a lower rate of technical complications and post-operative dislocation in octogenarians [16].

For rTKA, octogenarians have higher rates of blood transfusion and slightly longer length of stay compared to younger populations but there were no differences in mortality or readmission [14]. For octogenarians, ROM was improved after an rTKA, though not to the same extent as octogenarians undergoing primary procedures [15]. Further, no difference in systemic complications between primary and revision TKA was found in this age group, and the 5-year and 10-year lifetime survival rate was 82.1% and 42.2% for rTKA [15]. For both rTHA and rTKA, inpatient mortality rates did not exceed 4% among octogenarians [12,13].

In primary THAs and TKAs, patients with private payers were shown to have fewer complications and lower inpatient mortality rates compared to patients with government payers [18,19]. For rTKAs, Medicare insurance was found to be a risk factor correlated with increased readmission risk [20]. In our study, octogenarians and nonagenarians were more often insured by Medicare compared to sexagenarians, but lower than septuagenarians. This older population covered more often by Medicare might reflect the lower costs associated with revision procedures in this age group. Medicare may not fully reimburse younger patient revision procedures, and thus these patients might seek additional private insurers to cover costs. Future research investigating the types of insurance payers and secondary insurance among different age groups is needed to optimize the resource-allocation among elderly patients undergoing rTJAs.

Previous studies examining the costs of an rTJA in octogenarians and nonagenarians are limited. For rTKAs, octogenarians and nonagenarians have been shown to have total hospital charges of 86,203 USD and 98,828 USD, respectively [12]. For rTHAs, octogenarians and nonagenarians have been shown to have total hospital charges of 83,132 USD and 93,170 USD, respectively [13]. However, charges are prices listed by hospitals for claims purposes and do not represent the actual costs of care. Payers are unlikely to reimburse the full charge price and thus they represent a less direct method of understanding the expenses necessary for caring for these patients. Using TDABC, the gold-standard cost methodology for TJAs [21,22,23], we did not find any significantly different episode-of-care costs for rTKAs and rTHAs among octogenarians and nonagenarians as compared to sexagenarians or septuagenarians.

For both rTHAs and rTKAs, octogenarians and nonagenarians had a significantly longer length of stay, increased personnel costs from PACU through to discharge, and were more often discharged to skilled nursing facilities (SNFs) compared to the younger decade groups. Of note, the conservative Bonferroni-adjusted significance level of 0.003 to reduce the likelihood of a type I error was not reached for rTHA’s LOS and personnel costs. Despite this, the longer length of stay and increased personnel costs were likely related to more time-intensive nursing in PACU and on the floor due to comorbidities, and likely contributed to spending more time with physical therapists due to frailty. These physical therapy evaluations likely influenced their discharge disposition to locations other than home. All of these factors contributed to a higher total in-hospital cost. In order to measure the relative effect of these variables, we performed a multivariate linear regression model with standardized estimates. In our study, implant costs were shown to be the largest driver of in-hospital total costs. This is consistent with the literature showing that the TJA procedure implant price is the major driver of hospital costs [24,25,26].

Aside from the implant costs, the OR time, personnel cost from the PACU through to discharge, and procedure type were the most significant factors impacting the total in-hospital costs for revision procedures. Octogenarians and nonagenarians were found to have a decreased OR time compared to sexagenarians and septuagenarians for rTJAs overall. This was most likely a reflection of this patient group undergoing less complicated revisions, such as liner exchanges as opposed to full component exchange revisions. The decreased OR time had a significant effect on overall costs for these older patients, potentially offsetting the increased personnel costs needed for care. The impact of octogenarian and nonagenarian versus sexagenarian status was not associated with total costs after holding all other variables constant. This was consistent with our ANOVA results showing no difference in overall costs when comparing groups. The rTHA procedure type was associated with a significant decrease in overall costs, indicating that an rTHA is a less costly procedure than an rTKA among these groups.

A strength of the current study includes a relatively large sample size of 353 rTJA procedures at a single orthopedic institution over a two-year period. Further, our methodology of TDABC has been shown to be a more accurate cost account for TJAs than traditional methods [21,22]. The current study is not without limitations, including all limitations inherent to retrospective studies. Our analysis was dependent on administrative coding for the episode-of-care costs. However, minor errors in the coding methodology would be unlikely to affect the overall cost amounts. A high percentage of our oldest patient group underwent liner exchanges, representing less costly and time-consuming revisions, which influences the total in-hospital costs for this population. The authors believe that this represents a true distribution of the procedures for this age group and remains important for understanding costs in these patients when compared to other age groups. Our sample size for octogenarians and nonagenarians was only 59; however, this limited patient group represents a scarce study population and thus we believe this study represents an important model. Another limitation is that our cost methodology only captured costs through to discharge. The true cost of these procedures in this patient population includes their post-hospital expenses. A total of 42.8% of the octogenarians and nonagenarian patients needed to be discharged to an SNF, which has been shown to add 15,000 USD in post-acute care costs [27]. Additional healthcare expenditures to care for discharge disposition, readmissions, or complications are not captured in the current study and should be the focus of future research.

## 5. Conclusions

In conclusion, overall hospital costs for revision TJAs were not significantly more expensive for octogenarians and nonagenarians compared to sexagenarians and septuagenarians. As life expectancy increases, an increase in older patients needing revisions of primary TJA procedures is expected. Higher implant costs were seen to be a primary driver of total hospital costs for revision TJAs, regardless of age group. As the country focuses on value, which is defined as the outcomes per dollar spent, identifying episode-of-care costs in older patients undergoing revision TJAs is critical in delivering value-based health care.

## Figures and Tables

**Table 1:** Comparing patient characteristics for sexagenarians, septuagenarians, and octogenarians and older undergoing revision total hip or knee arthroplasty. ASA—American Society of Anesthesiologists classification, rTHA—revision total hip arthroplasty, rTKA—revision total knee arthroplasty.

Characteristic	Sexagenarians (*n* = 294)	Septuagenarians (*n* = 198)	Octogenarians and Nonagenarians (*n* = 59)	*p*-Value
Age (years), mean (SD)	64.61 (2.92)	73.74 (2.77)	84.64 (3.68)	<0.0001
ASA	2.3 (0.5)	2.4 (0.5)	2.7 (0.5)	0.0014
Procedure type				0.226
rTHA	46.3%	52.5%	59.3%	
rTKA	53.7%	47.5%	40.7%	
Insurance				<0.0001
Medicare	33.3%	75.8%	66.1%	
Private	66.7%	24.2%	33.9%	

**Table 2:** Comparing the outcomes and costs for revision total hip arthroplasties between sexagenarians, septuagenarians, and octogenarians and older. SNF—skilled nursing facility, PACU—post-anesthesia care unit. USD—United States dollar.

Variable	Sexagenarians (*n* = 136)	Septuagenarians (*n* = 104)	Octogenarians and Nonagenarians (*n* = 35)	*p*-Value
Discharge disposition				<0.0001
Home	85.3%	68.3%	34.3%	
Inpatient rehab	5.9%	11.5%	22.9%	
SNF	8.8%	20.2%	42.8%	
OR time (minutes)	199.8 (60.3)	189.7 (50.6)	172.3 (48.4)	0.0195
Length of stay (days)	2.8 (1.3)	2.7 (1.0)	3.6 (1.5)	0.0086
Personnel cost PACU through to discharge	-- USD	−1%	+22%	0.0064
Implant cost	-- USD	−4%	−4%	0.845
Total in-hospital cost	-- USD	−4%	−0.3%	0.618

**Table 3:** Comparing the outcomes and costs for revision total knee arthroplasties for sexagenarians, septuagenarians, and octogenarians and older. SNF—skilled nursing facility, PACU—post-anesthesia care unit. USD—United States dollar.

Variable	Sexagenarians (*n* = 158)	Septuagenarians (*n* = 94)	Octogenarians and Nonagenarians (*n* = 24)	*p*-Value
Discharge disposition				<0.0001
Home	85.5%	63.8%	16.7%	
Inpatient rehab	1.9%	8.5%)	8.3%	
SNF	12.6%	27.7%	75%	
OR time (minutes)	195.2 (50.4)	192.5 (50.6)	175.0 (53.1)	0.23
Length of stay (days)	2.8 (1.2)	3.4 (1.3)	3.8 (1.2)	<0.0001
Personnel cost PACU through to discharge	-- USD	+16%	+28%	0.0001
Implant cost	-- USD	+6%	−26%	0.214
Total in-hospital cost	-- USD	+6%	−14%	0.24

**Table 4:** Comparing the outcomes and costs for revision total hip and knee arthroplasties for sexagenarians, septuagenarians, and octogenarians and older. SNF—skilled nursing facility, PACU—post-anesthesia care unit. USD — United States dollar.

Variable	Sexagenarians (*n* = 294)	Septuagenarians (*n* = 198)	Octogenarians and Nonagenarians (*n* = 59)	*p*-Value
Discharge disposition				<0.0001
Home	85.4%	66.2%	27.1%	
Inpatient rehab	3.7%	10.1%	17%	
SNF	10.9%	23.7%	55.9%	
OR time (minutes)	197.3 (55.1)	191.1 (50.5)	173.4 (49.9)	0.0021
Length of stay (days)	2.8 (1.3)	3.1 (1.2)	3.7 (1.3)	<0.0001
Personnel cost PACU through to discharge	-- USD	+7%	+23%	<0.0001
Implant cost	-- USD	−1%	−24%	0.077
Total in-hospital cost	-- USD	+0.9%	−12%	0.185

**Table 5:** Multivariate linear regression results for the total in-hospital cost. Adjusted R-square = 0.9945. ASA—American Society of Anesthesiologists classification, PACU—post-anesthesia care unit.

Parameter	Standardized Estimate	95% Confidence Limits	*p*-Value
Implant cost	0.88389	0.87656–0.89123	<0.0001
OR time (minutes)	0.12140	0.11413–0.12868	<0.0001
Length of stay (days)	0.02504	−0.04027–0.09035	0.45
Personnel cost for the PACU through to discharge	0.11472	0.04880–0.18065	0.0007
Age group:septuagenarian	−0.00694	−0.01754–0.00367	0.19
Age group:octogenarian and nonagenarian	−0.00532	−0.01914–0.00850	0.45
Procedure type:rTHA	−0.03058	−0.04090–0.02026	<0.0001
Age	0.00160	−0.01279–0.01599	0.83
ASA	0.00293	−0.00322–0.00907	0.35
Discharge disposition: home	0.5771	−0.00545–0.00978	0.58
Discharge disposition: inpatient rehab	0.00432	−0.00231–0.01095	0.20

## Data Availability

Data sharing is not applicable to this article due to proprietary financial restrictions.
